# Persistence of Effects of VLBW/PT Birth Status and Maternal Emotional Availability (EA) on Child EA Trajectories

**DOI:** 10.3389/fpsyg.2018.02715

**Published:** 2019-01-29

**Authors:** Dale M. Stack, Célia Matte-Gagné, Daniel J. Dickson

**Affiliations:** ^1^Centre for Research in Human Development, Department of Psychology, Concordia University, Montréal, QC, Canada; ^2^School of Psychology, Laval University, Laval, QC, Canada

**Keywords:** very low birth weight (VLBW) preterm (PT), mother–child relationship, developmental patterns over time, socio-emotional development, longitudinal, adversity

## Abstract

Few studies have examined the longitudinal impact of birth status on the infant–mother relationship and on children’s socio-emotional development. In the present study we investigated developmental patterns of such relationships [using the Emotional Availability (EA) Scales] in fullterm and VLBW/PT infants from infancy to emerging school age. Our objectives were to: (a) model the developmental trajectories of EA dimensions (maternal sensitivity, structuring, non-hostility; child responsiveness, involvement) in a VLBW/PT and fullterm sample, (b) identify potential effects of VLBW/PT status on these trajectories, and (c) determine whether the effects of VLBW/PT status on children’s socio-emotional development (child EA) remained after accounting for the effect of maternal EA. Child–mother dyads (*n* = 109) were observed in home-based interactions (face-to-face and free play) when children were 6, 12, 18, and 57-months-old in fullterm (37–41 weeks, >2500 g; *n* = 48) and healthy VLBW/PT (26–32 weeks gestation, birth weight 800–1500 g, corrected for gestational age; *n* = 61) children. Developmental trajectories of maternal and child EA were assessed using multilevel growth modeling in Mplus. Results indicated that, even after controlling for maternal EA, there was a persistent negative effect of VLBW/PT birth status on child EA trajectories. Both initially and over time, VLBW/PT infants lagged behind their fullterm counterparts on levels of responsiveness and involvement with mothers. There was also a persistent positive effect of maternal EA (sensitivity and structuring) on child EA trajectories. Higher average levels of maternal sensitivity and structuring across time were also associated with higher and persistent levels of child responsiveness and involvement of their mothers. Importantly, results held after modeling both effects together, and after controlling for maternal education and child gender. Our results have implications for VLBW/PT children’s development, the parent–child relationship, and integrating family level factors and relationship dimensions in early prevention and intervention programs.

## Introduction

Biological birth status, and in particular premature birth, has long been considered of great consequence by developmental and health researchers and demonstrated to be a consequential risk factor for healthy development. Those children not only born preterm but very preterm (32 weeks’ gestation and less) and/or with a very low birth weight (VLBW; less than 1500 g) are considered at even higher risk for adverse and multiple short and long term developmental and behavioral outcomes (Tessier and Nadeau, 2007; Delonis et al., 2017; Zelkowitz, 2017; Scott et al., 2018). Improvements in medical technology and perinatal and intensive neonatal care have resulted in a growing number of children born very preterm and/or VLBW. As a result, critical questions have arisen related to the quality, stability, and patterns of developmental outcomes in these new biologically at-risk survivors. It is commonly accepted that most non-disabled survivors tend to experience motor and cognitive delays (Brydges et al., 2018), language delays (Zimmerman, 2018), and more “subtle” problems such as deficits in mathematics, reading, and spelling, attention and behavioral problems (e.g., Breeman et al., 2016; Scott et al., 2018), and deficits in executive functions (e.g., Brydges et al., 2018), which persist throughout childhood (e.g., Aarnoudse-Moens et al., 2009; Chan et al., 2016). In addition, these children continue to lag behind their peers as they transition into adulthood (e.g., Aarnoudse-Moens et al., 2009). The present study addresses an important gap in this literature by examining the early mother–child relationship and socio-emotional development of VLBW preterm child–mother dyads.

While abundant research attention has been devoted to cognitive-related processes and intellectual outcomes, less is known about VLBW preterm (VLBW/PT) children’s social and emotional development. Yet it appears that these children have difficulties in social adjustment and interactions with others and are generally less socially competent (Spittle et al., 2009; Zmyj et al., 2017). For example, they have been shown to have difficulties self-regulating and communicating (Nadeau et al., 2018). In addition, a few studies have examined emotion regulation strategies in VLBW/PT infant–mother dyads at 4 (Yaari et al., 2018), 6 (Jean and Stack, 2012), and up to 18 months (Atkinson et al., 2018), and results suggest subtle effects. There is also research that has examined neurodevelopmental vulnerabilities and parenting (e.g., sensitivity) as they relate to regulatory problems from birth to 18 months (Bilgin and Wolke, 2017). Despite a few studies having used short-term longitudinal designs and showed such effects as lower dyadic interaction quality (Delonis et al., 2017), little is known about the longitudinal socio-emotional processes and longer-term outcomes of these children; in particular, the impact of being VLBW/PT on socio-emotional development, how the mother–child relationship may influence such impact, the nature of the relationship patterns over time, and the persistence of any effects.

Yet establishing close relationships and connections with others promotes individual well-being (Emde and Spicer, 2000; Stack et al., 2012) while failure to do so can result in emotional and physical distress (Conger et al., 2000). Mother–child relationships form the foundation for children’s socio-emotional development and their future relationships. These positive relationships often foster resiliency and protect against adversities throughout children’s development (Musick et al., 1987; Luthar, 2006). However, a multitude of diverse conditions, including birth status (VLBW/PT), can threaten patterns of normative socio-emotional development and undermine the achievement of healthy outcomes later in life. For such at-risk children, the quintessential protective factor is a positive parent–child relationship, often with the child’s mother (Luthar, 2006; Barbot et al., 2014), although father–child relationships are also clearly important. Supportive relationships, positive parenting, and avoidance of the use of specific parenting behaviors that are dysfunctional have also been underscored as factors that enhance children’s adaptation (Luthar and Eisenberg, 2017). As a child develops, positive and reciprocal forms of emotional sharing are critical to the establishment and maintenance of healthy parent–child relationships (Biringen and Robinson, 1991; Aviezer et al., 1999; Bretherton, 2000; Lovas, 2005). In line with the Bioecological model (Bronfenbrenner and Morris, 2006), a consideration of contextual factors, including those at the family-level (in the present study, the relationship) are important to study and represent one layer of the larger system. Through this lens the importance of considering these relationship factors when studying the VLBW/PT child is highlighted, as these children may be more vulnerable to influences of family, demographics and environment (Giovannetti et al., 2013; Towers, 2018).

The mother–child relationship during development is therefore essential to consider in understanding the development of VLBW/PT children’s growth and how relationship dimensions (child and mother) may mitigate and protect against maladaptive development and behavior, socio-emotional and academic outcomes, and promote and build social competence and academic achievement within this at-risk population. VLBW/PT infants (and their mothers) may be at risk-for relationship problems, due in part to their vulnerability and fragility, length of hospitalizations, and specific behaviors. During social interactions, VLBW/PT infants are known to be less alert, more excitable, harder to soothe, and have poorer self-regulation (Jean and Stack, 2012; Provenzi et al., 2017) compared to fullterm infants. Thus, they are poor social partners, and often demonstrate fewer relationship building behaviors, including co-regulation (Doiron and Stack, 2017), making it potentially more difficult for mothers to engage with their infants optimally. Yet, the mother (and father) can be integral in mediating and fostering their social development (e.g., Montagna and Nosarti, 2016; Zmyj et al., 2017) through the frequent interactions that take place as their relationship develops. Because few studies have examined the longitudinal impact of birth status on the infant–mother relationship and because of the crucial value of this information for understanding developmental and adaptive functioning and targets for intervention, we investigated developmental patterns of such relationships and socio-emotional development [using the Emotional Availability (EA) Scales; Biringen et al., 2014] in fullterm and VLBW/PT children. We were particularly interested in the influence of VLBW/PT status on developmental trajectories in EA from infancy to emerging school age.

In considering relationships and relationship dimensions, a number of researchers have underscored maternal sensitivity as a protective factor against difficulties in the development of preterm infants (e.g., Faure et al., 2017; Neri et al., 2017; Provenzi et al., 2017; Zmyj et al., 2017). As a result, sensitivity is a variable that many believe should be measured as well as then linked to measure associations with children’s socio-emotional development. Parental structuring and/or scaffolding, as well as directive and non-directive guidance are also considered to be important parenting practices (Vygotsky, 1978; Blandon and Volling, 2008; Briscoe et al., 2017) linked to children’s socio-emotional outcomes. Similarly, the child’s responsiveness to the mother and the inherent reciprocity in healthy social exchanges and emotional development is another integral factor to consider (e.g., Biringen and Robinson, 1991; Aviezer et al., 1999; Bretherton, 2000; Lovas, 2005). Together with the aforementioned studies on social interaction and dyadic quality, findings highlight the crucial way with which the parent–child relationship is implicated in VLBW/PT infants’ social and emotional outcomes. However, no prior studies to our knowledge have examined developmental patterns of change in the mother–child relationship in fullterm and VLBW/PT child–mother dyads from infancy to emerging school age using a four-wave design, and none have examined the effects of being VLBW/PT on these trajectories over time.

The EA Scales are well-suited to capturing critical aspects (i.e., sensitivity) of the relationships between parents and their children, both early in a child life, and as they grow older (Biringen and Easterbrooks, 2012). Put simply, EA is a relational construct that encapsulates mothers’ and children’s ability to well-regulate their interactions (Emde, 1980; Emde and Spicer, 2000), while taking into account both partners’ behaviors (Biringen, 2000). By using a multidimensional framework, the EA scales measure (via dyadic observational codes) parent-specific (parent EA), and child-specific (child EA) interactive behaviors that are widely regarded as important indicators of socio-emotional development. A growing body of evidence shows that the EA Scales reflect key indicators of the quality of the parent–child relationship and the child’s socio-emotional development (child EA) (see 2012 special issue in Developmental and Psychopathology; for reviews see Biringen, 2000; Biringen and Easterbrooks, 2012; Biringen et al., 2014). In the present study, we focused on all dimensions of the EA Scales: young children’s EA as captured by two dimensions (i.e., responsiveness and involvement) and mothers’ EA as captured by three dimensions (i.e., sensitivity, structuring, and non-hostility) that are evaluated and coded observationally by the EA Scales (Biringen et al., 1998). Four of these five EA dimensions were measured at four occasions and one dimension (child involvement) at three occasions, from infancy to emerging school age in fullterm and VLBW/PT child–mother dyads; this enabled an examination of growth trajectories in the relationship and in socio-emotional development (child EA) over time in a group considered at-risk and born under adversity.

This examination of growth trajectories is both crucial and timely. A central goal in developmental research is to identify intra-individual and inter-individual developmental patterns and predictors of human development. Most of the studies that have examined this issue rely upon cross-sectional or two-wave designs that do not provide a sufficient basis for studying developmental patterns (Willett et al., 1998). Indeed, a limited number of studies have followed VLBW/PT children over time using a prospective longitudinal design with repeated measures. As such, due to this dearth of research, there is a limited understanding of the developmental trajectories of these children across time and the course and persistence of socio-emotional problems in this population. Considering the increasing rates of VLBW and PT births around the world, more multi-wave longitudinal studies examining the developmental trajectories of VLBW/PT children are needed. In the present longitudinal study with four measurement occasions embedded within a developmental framework, we examined intra- and inter-individual changes of an important aspect of the mother–child relationship and children’s socio-emotional development in fullterm and VLBW/PT child–mother dyads, by using a multilevel growth modeling approach (Hedeker, 2004; Burchinal et al., 2006).

Consistent with Cicchetti and colleagues’ developmental psychopathology framework (e.g., Barnett et al., 1993; Cicchetti, 2006; Cicchetti and Toth, 2009), to best understand the underlying mechanisms driving the appearance and maintenance of maladaptive and disordered behavior, and to identify ways to circumvent them, it is important to investigate all pathways to adaptive and maladaptive outcomes throughout development. To identify such pathways, developmental researchers are encouraged to examine risk and protective factors, often by analyzing associations across multiple levels so as to identify potential avenues for prevention and intervention for those most at risk for developing later disorders. Examining EA and the developmental trajectories in a sample including both fullterm and VLBW/PT child–mother dyads allows us to study a population varying in risk and provides an important means of understanding the pathways to adaptive and maladaptive outcomes. Both the Bioecological and the developmental psychopathology models framed the present study.

The primary objective of this study was to explore birth status as a risk factor for developmental patterns of change in the mother–child relationship and in child EA in a sample of typically developing fullterm and VLBW/PT children from infancy to emerging school age. The specific objectives of this study were threefold. As a needed intermediate step in investigating the effect of VLBW/PT status on EA, the first objective was to model and describe the developmental trajectories of five components of EA: maternal sensitivity, structuring and non-hostility, and child responsiveness and involvement, from infancy through emerging school age in a sample including both VLBW/PT and fullterm children. The second objective was to identify potential effects of being VLBW/PT on these trajectories. We anticipated that VLBW/PT status would have a negative effect on these trajectories, particularly on child EA trajectories, if effects were revealed. The third objective was to determine whether the effect of VLBW/PT status on children’s socio-emotional development (child EA) remained after accounting for a potential protective effect of maternal EA (a known predictor of children’s socio-emotional development; Matte-Gagné et al., 2018). The persistence of effects has rarely been examined, and never with child EA and VLBW/PT birth status.

A longitudinal design with four time points (three for child involvement) was used. The trajectories were explored using multilevel growth modeling. This growth modeling technique provides strong statistical methods that are useful in describing individual developmental patterns and determining their predictors (Burchinal et al., 2006). Critically, this technique also allows for the partitioning of mother–child associations into within-dyad and between-dyad components, thereby isolating each mother’s contribution to their specific child’s socio-emotional development, while also estimating the influence of birth status and other important characteristics (e.g., child gender, maternal education). Finally, we conducted this with a group of VLBW/PT infants who were intensively screened, all serious complicating medical conditions were ruled out (see section “Materials and Methods”), and who were corrected for gestational age. Consequently, results can be largely attributed to early birth and VLBW and not to complicating medical factors common in this population.

## Materials and Methods

### Participants

A total of 109 child–mother dyads were observed in interactions in their homes when children were approximately 6, 12, 18, and 57-months old. Following ethics review and approval (Hospital and University), 48 fullterm and 61 healthy VLBW/PT infants were recruited from a major community teaching hospital (Montréal, QC) at the same time and by the same research team for the present study in collaboration with the VLBW follow-up clinic and the chief Neonatologist. When VLBW/PT infants were between 3 and 4 months of age, nurses pre-screened the infants for medical issues and performed assessments of various health-related variables. Only those VLBW/PT infants who were healthy, were between 800 and 1500 g, and were living with their biological mothers, were included in the current study. We excluded infants who suffered from major illnesses or medical complications (e.g., retinopathy, hydrocephalus, neurological impairments, hearing problems, Grade IV intra-ventricular hemorrhage, etc.); infants with congenital abnormalities; infants who were frequently hospitalized since the neonatal period; infants of teenage (<18 years) or diabetic mothers; and infants with mothers who experienced a history of drug-abuse, mental illness, sexual assault, or inadequate prenatal care.

Mothers of VLBW/PT infants who were retained in the current study were informed of the current study’s purpose. If they were interested in participating, they were contacted by telephone and were asked to voluntarily participate. Fullterm dyads were recruited using birth records from the same hospital, and were limited to those dyads with infants of normal birth weight (>2500 g) who were born between 37 and 41 weeks of gestation and had medical histories with no major health complications. Qualifying dyads received a letter requesting their participation and were contacted in the same manner as the VLBW/PT dyads.

Data in the current study were collected at four different time points, based on the ages of the children. All VLBW/PT infants were corrected for gestational age. VLBW/PT infants were 5.75 months old at T1 (*SD* = 0.52), 12.59 months old at T2 (*SD* = 0.51), 18.55 months old at T3 (*SD* = 0.55), and 59.13 months old at T4 (*SD* = 8.24). Fullterm infants were 5.42 months old at T1 (*SD* = 0.24), 12.44 months old at T2 (*SD* = 0.48), 18.53 months old at T3 (*SD* = 0.56), and 56.45 months old at T4 (*SD* = 5.79). Table 1 provides information on demographic and medical variables for families with VLBW/PT and fullterm infants. At T1, *t*-tests revealed that mothers of fullterm children, when compared to mothers of VLBW/PT children, were somewhat younger [*t*(109) = 2.29, *p* = 0.024, *d* = 0.65; *M*_fullterm_ = 30.23 years; *M*_V LBW/PT_ = 32.64 years], and more educated [*t*(109) = 3.44, *p* = 0.001, *d* = 0.43; *M*_fullterm_ = 14.52 years; *M*_V LBW/PT_ = 13.11 years].

**Table 1:** Demographic and medical characteristics for fullterm and VLBW/PT infants.

	Fullterm (*n =* 48)		VLBW/PT (*n* = 61)	
	*M*	*SD*	*M*	*SD*
Maternal age (years)^∗^	30.22	5.01	32.63	5.58
Maternal education at birth^∗∗^	14.52	2.06	13.11	2.11
Infant birth weight (g)^∗∗∗^	3504	0.43	1097	0.27
Infant gestational age (weeks)^∗∗∗^	39.54	1.11	28.54	2.31
Emergency C-section (%)^∗∗∗^	35.00	0.63	0.80	0.51
1 min APGAR^∗∗∗^	8.63	0.97	5.80	2.24
5 min APGAR^∗∗∗^	9.19	0.54	7.80	1.43
Length of hospital stay (days)^∗∗∗^	3.69	4.08	64.15	30.50
Infant length at birth (cm)^∗∗∗^	50.67	4.42	37.37	3.43
Infant head circumference (cm)^∗∗∗^	35.00	1.56	26.51	2.39
Infant weight at 6 months (g)	6904	994	6662	1072
Infant height at 6 months (cm)^∗^	64.26	4.26	62.56	4.03
Infant age at 6 months (months and days)^∗∗∗^	5.42	0.24	5.74	0.51

*Mean differences between groups were evaluated using *t*-tests. ^∗^*p* < 0.05, ^∗∗^*p* < 0.01, ^∗∗∗^*p* < 0.001 (two-tailed).*

A small to moderate proportion of dyads did not participate at specific time points. For mother-VLBW/PT child dyads, 13 (21.3%) dropped out after T1, 4 (6.6%) dropped out after T2, 7 (11.5%) dropped out after T3. An additional 13 (21.3%) mother-VLBW/PT child dyads did not participate at one or two earlier time points, but had rejoined the study by T4. For mother-fullterm child dyads, 7 (14.6%) dropped out after T1, 4 (8.3%) dropped out after T2, 9 (18.8%) dropped out after T3. An additional 2 (4.2%) mother-fullterm child dyads did not participate at one or two earlier time points, but had rejoined the study by T4. In total, 26 (54.2%) mother-VLBW/PT child dyads and 24 (39.3%) mother-fullterm child dyads participated at all time points. An ANOVA did not reveal any significant differences on child EA at T1 depending on the number of waves of participation, or on its interaction with infants’ birth status.

### Procedure

As part of a larger study, the current study included a series of questionnaires, interviews, and naturalistic observations taking place in participants’ homes. The home visits were approximately 90 min and occurred only at times when the mother believed they were the best timing for their child (e.g., when the child was well-rested, well-fed, and was alert). At each time point in the current study, researchers visited participating families and explained the overall procedure to mothers, asked mothers to sign an informed consent form, and set up the camera and study materials. Next, researchers requested that mothers take part in a short interview and complete a series of questionnaires assessing family socio-demographics. Upon their completion (roughly 20–30 min later), if mothers and children felt comfortable, they were asked to interact and play with each other as they normally would.

At T1 (when infants were roughly 6 months old), mother–infant dyads participated in two separate 2-min videotaped sessions where each member interacted with each other face-to-face. Infants were placed in an infant seat in front of mothers. Each of the 2-min periods came before and after a 2-min still face period where mothers were intrusted to hold a ‘still face’ (Still-Face procedure; Tronick et al., 1978). For the current study, we rely on coded EA data observed during the first “Normal” face-to-face period. At T2 (when children were roughly 12 months old), T3 (18 months old), and T4 (57 months old), mother–child dyads were instructed to engage in a 15-min free-play task utilizing standardized (i.e., child age-appropriate) toys while being videotaped in a well-lit room with minimal distractions. These toys included puzzles, doll, building blocks, books, and a tea set. Neither experimenters nor other family members were allowed in the room while interactions were taking place.

### Measures

#### Demographic Information Questionnaire (DIQ)

Socio-demographic information was collected using the DIQ at T1 and included the following variables among others: mothers’ current age, mothers’ occupational status, mothers’ education, children’s age, and children’s gender. Mothers’ education was calculated by taking the maximum number of years of education for each participant, with values ranging from the completion of elementary school to the completion of college or university. Child gender was coded as 1 = *male*, 2 = *female*. The DIQ measure has proven effective in collecting participant demographics, and has been used in past studies (e.g., Serbin et al., 1998; De Genna et al., 2006; Briscoe et al., 2017).

#### EA Scales

To assess the quality of mother–child interactions, we coded dyadic interactions via the EA Scales (2^nd^ Ed; Biringen et al., 1988, 1993). At the onset of the study (T1), only the 2^nd^ edition of this measure was available. In order to maintain consistency in the manner EA was assessed, we retained this measure over all following time points (T2–T4). We assessed both mother and child dimensions of EA because both have been identified as important indicators of socio-emotional development. As such, the following dimensions were coded by observers during interaction sessions at each time point:

(1) *maternal sensitivity* to children’s emotional needs and cues (e.g., maternal behaviors which both reflect a clear understanding of the child’s emotions and provide an emotionally sensitive and developmentally appropriate response).
(2) *maternal structuring* of dyadic interactions as a function of the child’s emotional needs (e.g., maternal behaviors which establish and reinforce limits while following the child’s initiations).
(3) *maternal non-hostility* (e.g., maternal behaviors that are consistent with pleasantness, non-criticalness, patience, and that are non-rejecting or antagonistic).
(4) *child emotional and social responsiveness* to the mother (e.g., child behaviors that convey a willingness to engage with mothers, and expressions of clear enjoyment while doing so).
(5) *child involvement* (e.g., child behaviors which attend to, initiate, and are involving of mothers’ interactions in play).

Codings were on 5- or 9-point scales. For the current study, we inverted the maternal non-hostility scores and termed the scale “hostility” (as in Stack et al., 2012). The upper-end of these five scales respectively represent optimal levels of maternal sensitivity and structuring, overtly hostile behaviors, and optimal levels of child responsiveness and involvement. All EA Scales except child involvement were coded at all four time points. Child involvement was only first coded at T2 (12 months) consistent with the measure. We provide further detail on how this difference affects the current study’s design in the analytical plan below.

Past studies have demonstrated the validity of the EA Scales in measuring the EA of parents and children at different ages (Bornstein et al., 2012). While it is ideal to allow for longer periods of observation (Biringen et al., 2005), extensive research has reliably assessed EA in parent–child interactions using the EA scales in relatively short time periods (e.g., 5- to 15-min) across a wide range of contexts (for a review, see Biringen et al., 2014). Many studies have established the predictive and convergent validity of the EA scales; for example, EA scales have been found to associate with maternal depression (Easterbrooks et al., 2012), child attachment (Easterbrooks et al., 2012), adult attachment representations (Coppola et al., 2006), child emotion understanding (Garvin et al., 2012), family SES (Chaudhuri et al., 2009), child goal encoding (Licata et al., 2014), infant emotion regulation (Little and Carter, 2005), and infant sleep patterns (Scher, 2001). The EA construct and its scales have received abundant research attention with studies ranging across age (0–14 years) and in normative, clinical and high-risk populations (e.g., feeding disorders; intellectual disabilities; high-risk community sample; disadvantaged; drug exposed and depressed mothers; see Biringen et al. (2014). For a more detailed description of the EA Scales, see Biringen and Easterbrooks, 2012; Biringen et al., 2014).

After completing a 3-day training course on-site, an original set of coders was certified on the proper and reliable coding of the Biringen tapes. Subsequent training continued to be provided by our trained team. In coding the present data the same coders coded the EA of both mothers and children at all time points in order to limit the introduction of rater error to the intra-individual variability in EA across time. As a further safeguard, the coders were instructed to complete five coding passes for each video record – one for each of the five EA dimensions. At each time point, at least a quarter of the sample was randomly selected to be double-coded; reliability coefficients (ICCs; intra-class correlation coefficients) were acceptable, ranging between 0.82 and 0.99).

### Analytic Strategy

We conducted multilevel growth modeling analyses using *MPlus* (version 8.1) (Muthén and Muthén, 1998-2017) with maximum likelihood with robust estimators (MLR) to describe and predict intra-individual patterns of trajectories in mother and child EA. MLM was chosen given its ability to easily handle repeated measures of the same outcomes where time points moderately vary across participants (Singer and Willett, 2003; Burchinal et al., 2006). Multilevel growth modeling estimates inter-individual variability in intra-individual patterns of change over time by decomposing change over time into two levels: a Level 1 component which represents change across time in outcomes within the individual, and a Level 2 component which represents how such patterns of change differ between individuals in a sample. As an extension, this accommodates the inclusion of both time-invariant and time-variant predictors that might account for influences on initial levels and change across time. For each EA scale, we provide intra-class correlations coefficients (ICCs) that indicate the proportions of variances in EA that vary within individuals (across time) from total variances. These estimates are equivalent to within-person stabilities of EA.

#### Modeling Change in Maternal and Child EA Across Time

We followed a multistep procedure for estimating change in EA across time (Singer and Willett, 2003). Consistent with the first objective, we modeled intercepts (i.e., initial levels) and slopes (i.e., yearly rates of change – or trajectories) in the five dimensions of EA. We estimated three potential models: (a) one that estimated no over time change, (b) one that estimated linear change (defined individually in years), and (c) one that estimated both linear and non-linear change. To increase parsimony, only the best fitting models were selected. Non-significant (*p* > 0.05) parameters were then trimmed for the unconditional mean models (Model A). As previously noted, in contrast to the other four EA scales, which were assessed at all four time points (6 to 57 months), child involvement was assessed only over the latter three time points (12 to 57 months) consistent with the EA measure and how it is coded. While three data points still accommodate the estimation of linear growth curves, it should be noted that the interpretations regarding the intercepts and slopes for child involvement should be limited to the 12–57 months timespan.

#### VLBW/PT Status as a Predictor of EA Trajectories

Next, consistent with the second objective, once the overall trajectories were established, we investigated the extent to which VLBW/PT status explained the initial levels (intercepts) and trajectories (slopes) of EA. In order to reduce the likelihood that associations found in our model were due to confounding demographic variables, we controlled for child sex and maternal education, each taken from the DIQ measure. We selected these controls because education (as part of SES) and child gender have both been associated with family interaction processes (Conger and Donnellan, 2007; Zahn-Waxler et al., 2008), and there are more preterm births among boys (Zeitlin et al., 2002) and in low-SES families (Morgen et al., 2008). Three models were estimated. In the first model (Model B), VLBW/PT status, child sex, and maternal education were entered as predictors of the EA’s intercepts. In the second model (Model C), non-significant (*p* > 0.05) effects were trimmed from the prior model, and then the same variables were entered as predictors of EA’s slopes. In the third model (Model D), any remaining non-significant (*p* > 0.05) fixed effects were trimmed.

#### Maternal EA as Predictors of Child EA

Consistent with the third objective, we then entered maternal EA as predictors of the final child EA trajectories (previously presented in Model D). To this end, as recommended by Curran and Bauer (2011) we distinguished maternal EA’s between-dyad effects on child EA from their within-dyad effects. To account for between-dyad effects, we aggregated maternal sensitivity and structuring across all assessment waves, and entered them as mean-centered time-invariant predictors at Level 2. To account for within-dyad effects, we entered maternal sensitivity and structuring as person-mean centered time-variant predictors at Level 1. Maternal hostility was not entered as a predictor due to low variance (see below). Models were separately estimated for child responsiveness and child involvement. Only maternal EA data from T2, T3, and T4 were entered for child involvement because the latter was not assessed at T1. Model E describes the initial results, and Model F describes the results after non-significant fixed effects (*p* > 0.05) had been trimmed.

#### Handling of Missing Data

Missingness in the current study came from two-sources: attrition and wave-level missingness. Roughly 41% of the sample did not participate at one or more points in the study. A description of missingness at each wave for both groups can be found in Section “Participants.” To account for missing data, a total of 20 imputed datasets were generated from a Markov chain Monte Carlo simulation in *Mplus*. Final model estimates were derived from a meta-analysis of results from each dataset. Little’s test was not statistically significant, χ^2^(109) = 112.61, *p* = 0.39, therefore we treated the data as missing completely at random.

## Results

### Descriptive Statistics

Table 2 presents means, standard deviations, ranges, and correlations of maternal and child EA over all four time points of the study. Mothers demonstrated consistently high levels of sensitivity and structuring and consistently low levels of hostility over the course of the study. Child responsiveness and involvement were consistently moderately high over the course of the study. In terms of mean level changes over time, we found a minor increase in child responsiveness and involvement between the earliest time point (T1 or T2) and T4 (*t* = -4.34, *p* < 0.001 and *t* = -3.09, *p* = 0.003, respectively).

**Table 2:** Descriptive statistics and correlations across child age (relative stability) for the Emotional Availability (EA) Scales.

		Fullterm		VLBW/PT			Correlations across age (stability)		

EA dimensions	Age	*M*	*SD*	*M*	*SD*	Range	12	18	57
Sensitivity	6	7.90	0.86	7.53	1.00	3.00–9.00	0.17	0.42^∗∗^	0.36^∗∗^
	12	7.47	0.99	7.26	1.14	3.00–9.00	–	0.23^t^	0.25^∗^
	18	7.32	1.12	7.06	1.17	1.00–9.00	–	–	0.37^∗∗^
	57	7.44	0.91	7.01	1.03	5.00–9.00	–	–	–
Structuring	6	4.44	0.56	4.32	0.69	2.00–5.00	0.19	0.28^∗∗^	0.21^t^
	12	4.37	0.60	4.13	0.77	3.00–5.00	–	0.29^∗∗^	0.34^∗∗^
	18	4.19	0.67	4.02	0.77	2.00–5.00	–	–	0.24^∗^
	57	4.33	0.69	3.87	0.81	2.00–5.00	–	–	–
Hostility	6	1.02	0.14	1.10	0.42	2.00–5.00	0.38^∗^	0.39^∗∗^	0.33^t^
	12	1.12	0.32	1.18	0.60	1.00–5.00	–	0.68^∗∗^	0.41^∗^
	18	1.08	0.23	1.20	0.63	1.00–5.00	–	–	0.47^∗∗^
	57	1.16	0.42	1.15	0.41	1.00–3.00	–	–	–
Child responsiveness	6	4.90	1.65	4.90	1.38	1.00–7.00	0.11	0.09	0.11
	12	5.81	0.95	4.99	1.36	2.00–7.00	–	0.34^∗∗^	0.27^∗^
	18	5.59	1.08	5.06	1.12	2.00–7.50	–	–	0.21^t^
	57	6.13	0.98	5.59	1.14	3.00–7.00	–	–	–
Child involvement	12	5.42	1.12	4.50	1.48	2.00–7.00	–	0.33^∗∗^	0.23^t^
	18	5.26	1.32	4.61	1.45	1.00–7.00	–	–	0.25^∗^
	57	5.83	1.26	5.34	1.53	3.00–7.00	–	–	–

*N = 48 fullterm and 61 VLBW/PT children. Ages are in months. ^*t*^*p* < 0.10, ^∗^*p* < 0.05, ^∗∗^*p* < 0.01 (two-tailed).*

A series of chi-square difference tests investigated whether the means of each EA scale significantly differed between mother-VLBW/PT child and mother-fullterm child dyads. Estimates were pooled across the 20 imputed datasets. Results indicated that mothers of VLBW/PT children demonstrated significantly less sensitivity at T1 only, χ^2^(1) = 3.93, *p* = 0.047, and less structuring at T4 only, χ^2^(1) = 8.28, *p* = 0.004. There were no differences between the two groups on maternal hostility at any time points. VLBW/PT children were observed to be significantly lower on responsiveness at T2, T3, and T4, χ^2^(1) = 4.26–11.52, *p* = 0.001–0.039, lower on involvement at T2, χ^2^(1) = 11.35, *p* = 0.001, and marginally lower on involvement at T3, χ^2^(1) = 3.50, *p* = 0.061. Taken together, these analyses provided preliminary evidence of initial, and in some cases, persistent and ongoing gaps in EA between mother-VLBW/PT child and mother-fullterm child dyads. However, growth curve analyses are better suited for detecting ongoing and stable differences between groups on maternal and child EA. These are described below.

### Growth Curves in Maternal and Child EA

Consistent with the first study objective, multilevel modeling (MLM) was conducted to estimate the developmental trajectories of the five components of EA. First, we estimated whether there was enough evidence of variation in trajectories of EA to justify more complex analyses.

Results of the unconditional mean models (see Model A in Tables 3 through 6) revealed sufficient (*p* < 0.10) within-person variation, or change over time, on four out of the five EA scales; we did not find significant variation for maternal hostility, thereby excluding the scale from further analyses. Notably, we also found evidence of moderate stability in EA, as indicated by intra-class correlations coefficients (ICCs; proportions of within-person variation to total variation) ranging from 0.16 to 0.34.

**Table 3:** The growth models of maternal sensitivity between 6 and 57 months.

ICC = 0.29			Model A	Model B	Model C	Model D
**Fixed effects**						
Initial status	Intercept	γ_00_	7.60*** (0.09)	7.60*** (0.08)	7.61*** (0.09)	7.60*** (0.09)
	Maternal education	γ_01_		0.11** (0.03)	0.11** (0.04)	0.12*** (0.03)
	Sex (being a girl)	γ_02_		0.12 (0.14)		
	VLBW/PT status	γ_03_		–0.15 (0.14)		
Rate of change	Intercept	γ_10_	–0.36** (0.12)	–0.37** (0.12)	–0.40** (0.13)	–0.36** (0.12)
	Maternal education	γ_11_			0.02 (0.06)	
	Sex (being a girl)	γ_12_			–0.02 (0.21)	
	VLBW/PT status	γ_13_			0.09 (0.24)	
Quadratic rate of change	Intercept	γ_20_	0.06* (0.03)	0.06* (0.03)	0.07** (0.03)	0.06* (0.03)
	Maternal education	γ_21_			0.00 (0.01)	
	Sex (being a girl)	γ_22_			0.01 (0.05)	
	VLBW/PT status	γ_23_			–0.04 (0.06)	
**Variance/residual variance components**						
Level 1	Within-person	$\sigma_{E}^{2}$	0.79*** (0.13)	0.79*** (0.13)	0.78*** (0.13)	0.79*** (0.13)
Level 2	In initial status	$\sigma_{0}^{2}$	0.32** (0.09)	0.24** (0.08)	0.25** (0.08)	0.25** (0.08)
Goodness-of-fit	LL		–621.30	–611.92	–610.31	–613.21
	AIC		1252.60	1239.84	1244.61	1238.43
	BIC		1272.99	1272.46	1293.55	1262.89

*N = 109. Standard errors are within parentheses. Rate of change is estimated in years. VLBW/PT, very low birth weight/preterm; LL, log likelihood; AIC, Akaike Information Criterion; BIC, Bayesian Information Criterion. ^∗^*p* < 0.05, ^∗∗^*p* < 0.01, ^∗∗∗^*p* < 0.001.*

We also estimated the intercepts and slopes of mean-levels of EA. Results are presented below.

#### Maternal EA

As seen in Model A in Tables 3, 4 the means of the intercepts of maternal sensitivity and structuring (γ_00_ = 7.60 and 4.28) and their variances ($\sigma_{0}^{2}$ = 0.32 and 0.13) were statistically significant. In terms of slopes, sensitivity non-linearly decreased over time (γ_10_ = -0.36 and γ_20_ = 0.06), and structuring decreased linearly over time (γ_10_ = -0.06). All variances in the rates of change for sensitivity and structuring, and their covariances with their respective intercepts, were non-significant and were trimmed.

**Table 4:** The growth models of maternal structuring between 6 and 57 months.

ICC = 0.26			Model A	Model B	Model C	Model D
**Fixed effects**						
Initial status	Intercept	γ_00_	4.28*** (0.05)	4.28*** (0.05)	4.28*** (0.05)	4.28*** (0.05)
	Maternal education	γ_01_		0.08** (0.03)	0.09** (0.03)	0.08** (0.02)
	Sex (being a girl)	γ_02_		0.07 (0.09)		
	VLBW/PT status	γ_03_		–0.12 (0.09)		
Rate of change	Intercept	γ_10_	–0.06* (0.02)	–0.06* (0.02)	–0.05* (0.02)	–0.05* (0.02)
	Maternal education	γ_11_			–0.01 (0.01)	
	Sex (being a girl)	γ_12_			0.01 (0.04)	
	VLBW/PT status	γ_13_			–0.08* (0.04)	–0.08* (0.04)
**Variance/residual variance components**						
Level 1	Within-person	$\sigma_{E}^{2}$	0.39*** (0.04)	0.39*** (0.04)	0.39*** (0.04)	0.39*** (0.04)
Level 2	In initial status	$\sigma_{0}^{2}$	0.13** (0.04)	0.09** (0.03)	0.09** (0.03)	0.09** (0.03)
Goodness-of-fit	LL		–461.81	–449.90	–447.63	–448.31
	AIC		931.61	913.80	911.26	908.61
	BIC		947.92	942.35	943.88	933.08

*N = 109. Standard errors are within parentheses. Rate of change is estimated in years. VLBW/PT, very low birth weight/preterm; LL, log likelihood; AIC, Akaike Information Criterion; BIC, Bayesian Information Criterion. ^∗^*p* < 0.05, ^∗∗^*p* < 0.01, ^∗∗∗^*p* < 0.001 (two-tailed).*

#### Child EA

As seen in Model A in Tables 5, 6 the means of the intercepts of child responsiveness and child involvement (γ_00_ = 5.08 and 4.76) and their variances ($\sigma_{0}^{2}$ = 0.27 and 0.55) were statistically significant. In terms of slopes, both responsiveness and involvement linearly increased over time (γ_10_ = 0.18 for both). All variances in the rates of change for child responsiveness and involvement, and their covariances with their respective intercepts, were non-significant and were trimmed.

**Table 5:** The growth models of child responsiveness between 6 and 57 months.

ICC = 0.16			Model A	Model B	Model C	Model D
**Fixed effects**						
Initial status	Intercept	γ_00_	5.08*** (0.10)	5.08*** (0.10)	5.08*** (0.10)	5.08*** (0.10)
	Maternal education	γ_01_		0.07 (0.04)		
	Sex (being a girl)	γ_02_		0.14 (0.16)		
	VLBW/PT status	γ_03_		–0.37* (0.16)	–0.39* (0.19)	–0.48** (0.15)
Rate of change	Intercept	γ_10_	0.18*** (0.04)	0.18*** (0.04)	0.18*** (0.04)	0.18*** (0.04)
	Maternal education	γ_11_			0.02 (0.02)	
	Sex (being a girl)	γ_12_			–0.01 (0.06)	
	VLBW/PT status	γ_13_			–0.03 (0.08)	
**Variance/residual variance components**						
Level 1	Within-person	$\sigma_{E}^{2}$	1.35*** (0.14)	1.35*** (0.14)	1.34*** (0.14)	1.35*** (0.14)
Level 2	In initial status	$\sigma_{0}^{2}$	0.27* (0.13)	0.19^t^ (0.10)	0.20^t^ (0.11)	0.21^t^ (0.12)
Goodness-of-fit	LL		–715.70	–707.43	–707.92	–710.21
	AIC		1439.41	1428.86	1431.85	1430.41
	BIC		1455.72	1457.41	1464.47	1450.80

*N = 109. Standard errors are within parentheses. Rate of change is estimated in years. VLBW/PT, very low birth weight/preterm; LL, log likelihood; AIC, Akaike Information Criterion; BIC, Bayesian Information Criterion. ^*t*^*p* < 0.10, ^∗^*p* < 0.05, ^∗∗^*p* < 0.01, ^∗∗∗^*p* < 0.001 (two-tailed).*

**Table 6:** The growth models of child involvement between 6 and 57 months.

ICC = 0.34			Model A	Model B	Model C	Model D
**Fixed effects**						
Initial status	Intercept	γ_00_	4.76*** (0.15)	4.76*** (0.15)	4.76*** (0.15)	4.76*** (0.15)
	Maternal education	γ_01_		0.09 (0.06)		
	Sex (being a girl)	γ_02_		0.11 (0.22)		
	VLBW/PT status	γ_03_		–0.56* (0.23)	–0.86** (0.29)	–0.70** (0.22)
Rate of change	Intercept	γ_10_	0.18** (0.06)	0.18** (0.06)	0.18** (0.06)	0.18** (0.06)
	Maternal education	γ_11_			0.02 (0.02)	
	Sex (being a girl)	γ_12_			0.03 (0.07)	
	VLBW/PT status	γ_13_			0.11 (0.11)	
**Variance/residual variance components**						
Level 1	Within-person	$\sigma_{E}^{2}$	1.50*** (0.21)	1.50*** (0.21)	1.50*** (0.20)	1.50*** (0.21)
Level 2	In initial status	$\sigma_{0}^{2}$	0.55** (0.19)	0.38* (0.17)	0.39* (0.17)	0.42* (0.18)
Goodness-of-fit	LL		–569.73	–560.01	–560.67	–562.76
	AIC		1147.47	1134.02	1137.33	1135.52
	BIC		1162.63	1160.55	1167.65	1154.47

*N = 109. Standard errors are within parentheses. Rate of change is estimated in years. VLBW/PT, very low birth weight/preterm; LL, log likelihood; AIC, Akaike Information Criterion; BIC, Bayesian Information Criterion. ^∗^*p* < 0.05, ^∗∗^*p* < 0.01, ^∗∗∗^*p* < 0.001 (two-tailed).*

### Associations Between VLBW/PT Status and EA

Consistent with the second study objective, we estimated the effect of VLBW/PT status on the developmental trajectories (the intercepts and the slopes) of EA (see Model D in Tables 3 through 6).

Results indicated that VLBW/PT status was significantly associated with the intercepts, but not the slopes, of child responsiveness and involvement (γ_03_ = -0.48 and -0.70, *p* < 0.01). VLBW/PT children were observed to have lower stable levels of responsiveness and involvement when compared to their fullterm counterparts. Given that there were no effects of VLBW/PT status on the slopes of child responsiveness and involvement, both VLBW/PT and fullterm children linearly increased in child responsiveness and involvement at similar rates. As such, we find clear evidence that VLBW/PT children do not overcome their earlier initial with fullterm children.

Even after controlling for positive associations between maternal education and maternal EA (γ_01_ = -0.08, *p* < 0.05 for both sensitivity and structuring), VLBW/PT status was negatively associated with the linear slope of maternal structuring (γ_13_ = -0.08, *p* < 0.05). Follow up analyses revealed that only mothers of VLBW/PT children significantly decreased in structuring over time (slope = -0.08, *p* = 0.002 vs. slope = -0.01, *p =* 0.69 for fullterms).

### Maternal EA as Predictors of Child EA

Consistent with the third study objective, we fit a model that allowed for the estimation of associations between maternal and child EA, then we determined if effects of VLBW/PT status remained after controlling for these associations (see Model F in Table 7).

**Table 7:** Final models for child responsiveness and child involvement.

			Child Responsiveness		Child Involvement	
			
			Model G	Model H	Model G	Model H
**Fixed effects**						
Initial status	Intercept	γ_00_	5.16*** (0.09)	5.16*** (0.10)	5.07*** (0.13)	5.07*** (0.13)
	VLBW/PT status	γ_03_	–0.23* (0.12)	–0.25* (0.12)	–0.40* (0.18)	–0.40* (0.18)
	Sensitivity mean	γ_04_	0.61*** (0.16)	0.77*** (0.11)	0.40* (0.17)	0.40* (0.17)
	Sensitivity variation	γ_20_	0.29** (0.09)	0.29** (0.09)	0.38*** (0.10)	0.38*** (0.10)
	Structuring mean	γ_05_	0.21 (0.18)		0.50** (0.17)	0.50** (0.17)
	Structuring variation	γ_30_	0.33*** (0.08)	0.33*** (0.08)	0.41*** (0.12)	0.41*** (0.12)
Rate of change	Intercept	γ_10_	0.21*** (0.03)	0.22*** (0.04)	0.19*** (0.04)	0.19*** (0.04)
**Residual variance components**						
Level 1	Within-person	$\sigma_{E}^{2}$	1.13*** (0.12)	1.14*** (0.12)	1.18*** (0.16)	1.18*** (0.16)
Level 2	In initial status	$\sigma_{0}^{2}$	0.01 (0.05)	0.02 (0.05)	0.18 (0.11)	0.18 (0.11)
Goodness-of-fit	LL		–1630.86	–1632.45	–1207.37	–1207.37
	AIC		3287.72	3288.90	2444.74	2444.74
	BIC		3340.73	3337.83	2501.59	2501.59

*N = 109. Associations between the maternal EA (sensitivity and structuring) and the rate of change of child responsiveness and involvement were non-significant and were trimmed. Standard errors are within parentheses. Rate of change is estimated in years. VLBW/PT, very low birth weight/preterm; LL, log likelihood; AIC, Akaike Information Criterion; BIC, Bayesian Information Criterion. ^∗^*p* < 0.05, ^∗∗^*p* < 0.01, ^∗∗∗^*p* < 0.001 (two-tailed).*

As expected, results revealed the presence of significant positive associations between maternal and child EA at the within-dyad and between-dyad levels. At the within-dyad level, higher levels of maternal sensitivity and structuring at one time point predicted higher levels of child responsiveness (γ_20_ = 0.29 and γ_30_ = 0.33, *p <* 0.01) and involvement (γ_20_ = 0.38 and γ_30_ = 0.41, *p <* 0.001) at the same time point. At the between-dyad level, the intercepts of maternal sensitivity were positively associated with the intercepts of child responsiveness (γ_04_ = 0.77, *p <* 0.001) and involvement (γ_04_ = 0.40, *p <* 0.05). The intercepts of maternal structuring were only positively associated with the intercepts of child involvement (γ_05_ = 0.50, *p <* 0.01).

Importantly, independent of positive associations between maternal and child EA, VLBW/PT status continued to be negatively associated with the intercepts of child responsiveness (γ_03_ = -0.25, *p =* 0.04), and child involvement (γ_03_ = -0.40, *p =* 0.03). Taken together, even after accounting for positive within-dyad and between-dyad relationships between maternal and child EA, VLBW/PT children demonstrate lower estimated mean trajectories of child responsiveness and involvement when compared to fullterm children. Because there were no differences in the rates of change between the two groups, VLBW/PT children never overcame their initial gaps with fullterm children. Together, this pattern of results indicates that children who experienced consistently higher levels of maternal structuring and sensitivity over the course of the study, and children who were born fullterm, were both more responsive and involving of their mothers (and this effect persisted over time) compared to children who consistently experienced consistently lower levels of sensitivity and structuring or being VLBW/PT.

## Discussion

The effects of VLBW/PT status on the quality of the child–mother relationship and children’s socio-emotional development were assessed for the first time using an advanced growth modeling approach. This was an original direction as no studies to our knowledge have examined these effects of VLBW/PT children using a four-wave design from infancy to emerging school age. The findings have important implications for researchers, clinicians and health practitioners, and parents of preterm infants, as well as for prevention and intervention programs.

The principal results from our study’s growth curve analyses indicate that, even after controlling for maternal EA, there was a persistent negative effect of VLBW/PT birth status on child EA trajectories, i.e., responsiveness and involvement. After 6 months of age, VLBW/PT infants lagged behind their fullterm counterparts on their levels of responsiveness and involvement with their mothers. These findings remained even after accounting for the effects of maternal sensitivity and structuring over time. When we tested for mean level changes there was evidence for linear increases in means of responsiveness and involvement for both fullterm and VLBW/PT children, but the VLBW/PT children still continued to lag behind the fullterm children. Due to the fact that the effects persisted when maternal variables were controlled for in the models, we can be fairly confident that maternal EA are not responsible for this lagged difference in the two groups.

These results are certainly consistent with other studies that show effects of prematurity in cognitive, motor, emotion regulation, executive functioning, and other domains that continue in these children (e.g., Aarnoudse-Moens et al., 2009; Chan et al., 2016; also refer to introduction). However, studies investigating the persistent effect of preterm birth require repeated measures of the same variables and these are not common. In fact, there is a noticeable absence of long-term follow-up studies of preterm infants, particularly healthy VLBW/PT and specifically studies that address relationship and socio-emotional processes. This is the case in both clinical settings and in empirical research. Typically, follow-up studies are conducted on the groups of infants that are not healthy (or mixed groups) and are largely focused on physical development and cognitive outcomes (as opposed to psychological or developmental follow-ups). These generally take place in medical settings and not in the home environments. Thus, less is known about the psychological and socio-emotional development of healthy preterm infants and their relationships with their parents at home and little is known about developmental trajectories over time.

Our primary study variables were the mother–child relationship, and in particular two child EA dimensions as indicators of socio-emotional development (responsiveness and involvement), suggesting that it may be social interaction behavior that is at the core of our findings. Briefly, emotional and social *responsiveness* to the parent is measured through observing a willingness to engage and active engagement and positive response and enjoyment in the interaction. *Involvement* includes paying attention to mothers, and encouraging their involvement in play sessions (for a more detailed description of the EA Scales, see Biringen and Easterbrooks, 2012; Biringen et al., 2014). Both dimensions are important indicators of socio-emotional development and both embrace an active participation and initiative on the part of the child. Our results indicate that low child EA, reflected in responsiveness and involvement of their mothers may be a risk factor for VLBW/PT children. Our findings are consistent with those of others that indicate that VLBW/PT children have difficulties in socio-emotional domains relative to their fullterm peers (e.g., Doiron and Stack, 2017; Zmyj et al., 2017; Nadeau et al., 2018). They have been demonstrated to have more limited regulatory skills (e.g., Jean and Stack, 2012; Yaari et al., 2018), show more distancing and social monitoring (Montirosso et al., 2010), and rely more on their mothers in a reunion period following the still-face (Jean and Stack, 2012). These results suggest that preterm infants rely less on their own self-regulatory abilities and more on their mothers than their fullterm counterparts. However, these latter studies were not repeated measures designs over four waves and did not examine persistence of these effects. In a short-term longitudinal study focusing on the second year of life and play at 18, 24, and 30 months, Salvatori et al. (2016) found that responsiveness and involvement improved over time and global EA was lower in the preterm groups. However, they did not find differences between ELBW, VLBW, and fullterm on any of the dimensions of EA; notably these groups were tested individually and their sample had participated in a parenting intervention prior. Similarly, Matte-Gagné et al., 2018 showed an increase in child EA across infancy, however their sample was not preterm or VLBW. Finally, in a longitudinal study from 7 to 11 years Nadeau et al., 2018 showed increasing levels of victimization and social isolation in preterm children at 11 years leading to their argument for social marginalization, and underscoring that there are longer term problems in social functioning in children born premature that warrant further investigation.

Emotional and social competencies are considered central to school readiness, early school success, academic behaviors and achievement, and are also associated with attitudes toward learning and positive adjustment (Denham et al., 2016; Pekrun et al., 2017). Social interaction and interpersonal skills are a key component of these competencies; thus our results raise cause for concern. Given that parents and caregivers are central socializing agents they are pivotal in impacting these skills through their relationship with their child, sensitivity to cues and parenting strategies, and such means as play, social exchanges, modeling and demonstrations, as well as feedback and validation. Targeting them in interventions and public health initiatives, as well as pre-natal classes and post-natal well baby clinics are important directions toward assuring timely dissemination, awareness, and ultimately for enhancing these skills in VLBW/PT children.

Complementing our study’s contribution is that these results were demonstrated in a healthy VLBW/PT group and by measuring developmental patterns in relationship quality and two indicators of child socio-emotional development for the first time over four waves. That is, the conservative nature of our VLBW/PT group and the correction for gestational age largely suggest that our results are attributable to early birth and VLBW and not confounded by other medical or perinatal status variables.

Beyond the principal findings associated with the central focus of our paper, that of the effect of VLBW/PT status, there were several additional findings. Decreases in sensitivity and structuring were demonstrated however there was also a persistent positive effect of maternal EA (sensitivity and structuring) on child EA trajectories. That is, both within-dyad and between-dyad effects for maternal sensitivity and structuring were found. Regarding the former, higher levels of each at a given time point predicted higher levels of concurrent child responsiveness and involvement with mothers. Regarding the latter, across time the consistent mean levels of maternal sensitivity and maternal structuring predicted higher consistent levels of child responsiveness and child involvement with mothers. That is, children of mothers who expressed a higher average level of maternal sensitivity and structuring more often expressed a higher average level of responsiveness and were consistently more involving of their mothers across the preschool years, relative to those receiving lower maternal EA. Importantly, results held after modeling both effects together, and after controlling for maternal education and child gender. We also found that, for VLBW/PT children, maternal structuring linearly decreased over time whereas it remained stable for fullterm children. Such decreases may suggest that parenting stress associated with raising a VLBW/PT infant may result in disengagement in structuring behaviors over time. Alternatively, the decreases in structuring may suggest that because VLBW/PT infants are less engaged and involved in their interactions, mothers become less involved in the interactions over time, or less implicated in the play interaction.

Maternal sensitivity has long been considered and demonstrated to be a critical parenting dimension and has been linked to children’s socio-emotional outcomes (Bohr et al., 2018). Similarly, structuring (or scaffolding; Vygotsky, 1978) is also considered a key parenting dimension, has a long history, and is integrated in many parenting studies including those with a teaching, cognitive stimulation, or learning component (e.g., Saltaris et al., 2004; Briscoe et al., 2017). Recently in a sample of fullterm and preterm infants, Gueron-Sela et al. (2015) showed that preterm infants had poorer cognitive outcomes at 12 months when their parents used lower levels of co-parental structuring at 6 months. However, a moderating effect of temperamental reactivity was also revealed. Together with the present findings, there is evidence to support parenting and relationship factors as positive, and in some cases protective, in that they aid in contravening adversity (Fritz et al., 2018).

Finally, across groups, mothers with more education were observed to be consistently higher in sensitivity and structuring. This is in line with past findings concerning parenting, and is suggestive of higher-level and greater breadth of knowledge bases on parenting, development and developmental expectations. In a study of long-term cognitive outcomes of ELBW children, low maternal education was associated with poorer outcomes (Voss et al., 2012 as cited in Pelc and Gajewska, 2018). However, it is important to underscore that those with lower levels of education in our current study sample were not poor on these EA dimensions. Our findings suggest that VLBW/PT children have a specific vulnerability for socio-emotional difficulties and may require increased monitoring regarding their socio-emotional development even if they are healthy and growing up in a highly educated and sensitive family environment.

Consistent with the developmental psychopathology and bioecological models, our findings underscore the importance of examining early adverse experiences (VLBW and prematurity) in at-risk populations in prospective, longitudinal designs that include multiple levels over time in order that we may more deeply understand adversity, development, and those factors that may mitigate such adversity. Early care experiences and the quality of the mother–child relationship are known to be positive and these can potentially enable and foster change and adaptive outcomes in VLBW/PT children’s socio-emotional competencies where effects persist, such as responsiveness and involvement, if directly targeted. Promoting a nurturing, supportive, and caring environment early on is generally indicated (e.g., Dilworth-Bart et al., 2018).

The contributions of the present study are best evaluated within the context of several limitations and the need for further research. The nature of the four-wave prospective longitudinal design and the analysis of EA trajectories were strengths. Expanding the longitudinal design into middle and later childhood would enrich our understanding. Integrating additional variables that may mediate, or contextual factors that may contribute to the trajectories would also be an important step. Future studies should link relationship and socio-emotional variables over time to child outcomes in later childhood and adolescence and examine additional individual-level and family variables such as parental stress, mental health, and psychopathology. While studies in the broad cognitive domain have shown that deficits persist, even here the developmental pathways to adult outcomes are not clear and a number of variables likely play important roles in influencing these pathways and outcomes; these include genetic susceptibility, and environmental sources such as family and social support as moderators (Taylor, 2017). Mother, child, and contextual factors are likely to create and explain variations in the over time trajectories of maternal EA (Stack et al., 2012; Matte-Gagné et al., 2018). Worth noting is that mothers were the participants in the present study. Fathers are also an integral part of families and their role in building the relationship and influencing children’s trajectories is warranted. Finally, in our sample maternal education was positively associated with maternal EA, but also differed between the two birth status groups. As such, we cannot rule out the possibility that positive associations between birth status and maternal EA may be a function of maternal education, or related factors (e.g., conscientiousness, SES, stress, etc.). For example, women who are less diligent or organized may attain less education and may be more likely to struggle when raising a VLBW/PT child. However, future studies are warranted to further examine such possibilities using different methodologies to disentangle the effects of maternal education and birth status.

Taken together our results have implications for integrating the parent–child relationship and early interactions, as well as dimensions of the child’s socio-emotional competencies, in interventions and intervention programs. Importantly, given the persistence of the effects, this should take place early. While there have been some studies showing intervention effects with prematurity on various domains (Spittle et al., 2015; Evans et al., 2017; Faure et al., 2017; Fritz et al., 2018) most have focused on cognitive and motor outcomes (see also Spittle et al., 2015, for a systematic review) and few on domains relating to socio-emotional processes and competencies (Towers, 2018). A few exceptions exist (e.g., Wu et al., 2016 with a family centered RCT on emotion regulation; Fitzgerald’s, 2017 commentary on the same study). Notably it is also difficult to compare intervention programs and their effects given the wide variation in program elements, foci and gestational age (Spittle et al., 2015). However, what does seem to reliably surface are parenting and family-level factors that may contravene adversity (e.g., Zmyj et al., 2017; Fritz et al., 2018). Additional research is essential in order that targeting the key variables in prevention and intervention can be furthered.

## Conclusion

In a longitudinal design that measured relationship variables beginning in infancy through early childhood, VLBW/PT birth status and low child EA were risk factors for children’s socio-emotional development. As underscored, our results convey important implications for the design of preventive efforts and interventions, and for ensuring the integration of key child and parent variables into existing interventions. Moreover, there are implications for pre- and post-natal follow-ups, parenting interventions, and health and social policy. Building positive relationships and enhancing social and interpersonal skill sets for at-risk infants are central to enriching less adaptive relationships and to fostering resilient families. Ultimately, these are integral to supporting better futures for our children.

## Ethics Statement

This study was carried out in accordance with the recommendations of Concordia University Human Research Ethics Committee and the IRB/HREC of the major teaching hospital. All parents provided written informed consent for themselves and their children in accordance with the Declaration of Helsinki. The protocol was approved by the Human Research Ethics Committees at Concordia University and the Hospital.

## Author Contributions

DS contributed to the conception and design of the study, interpreted its findings, and wrote multiple drafts of the manuscript. CM-G and DD contributed to the statistical analysis, the interpretation of the study’s findings, and wrote sections of the manuscript. All authors read and approved the submitted version.

## Conflict of Interest Statement

The authors declare that the research was conducted in the absence of any commercial or financial relationships that could be construed as a potential conflict of interest.
